# Pacemapping

**Published:** 2005-01-01

**Authors:** Mauricio Moreno, Nicasio Perez-Castellano, Julian Villacastin

**Affiliations:** Unit of Arrhythmias, Cardiovascular Institute, San Carlos University Hospital, Madrid, Spain

Pacemapping (PM) is an electrophysiologic technique designed to help locating tachycardia sources by stimulating at different endocardial sites in order to reproduce the clinical tachycardia characteristics. A recorded electrocardiogram (ECG) during the clinical tachycardia has been conventionally used as reference. Yet, endocardial activation pattern during tachycardia may be utilized as well to guide the procedure. In focal tachycardia ablation, PM guide has consistently provided remarkable outcomes [1], while outcomes in reentrant tachycardia ablation are less favourable [2]

An important issue regarding PM is the electrical configuration of the stimulation catheter, bipolar or unipolar. During bipolar pacing, stimulation poles (anode and cathode) are located at the distal tip of the catheter and usually both are contacting the endocardium. Using this configuration, myocardium can be captured at the cathode, anode or both sites simultaneously decreasing mapping accuracy. On the contrary, unipolar pacing captures exclusively local myocardium next to the distal electrode [3]. Thus, unipolar pacing provides theoretical advantages over bipolar pacing by limiting the stimulated area. However, significant stimulation artefacts created by unipolar pacing limit its use. Bipolar pacing with minimally spaced electrodes constitutes an intermediate option capturing a limited myocardial area and inducing significantly less electrical artefacts.

Conduction properties of paced stimuli seem to depend on myocardial conditions. Pacing ventricular scar tissue, as in patients with prior myocardial infarction (MI), shows a delay between the electrical stimulus and the QRS onset, suggesting local slow conduction. These impaired conduction zones may represent potential substrates for reentrant circuits involved in ventricular tachycardia (VT).

Regarding atrial PM, it is unclear whether the conduction pattern of an atrial stimulus depend on the paced site, electrical voltage or the stimulating rate. This issue was recently addressed by Perez-Castellano el al [4]. Pacing at right and left atria, the authors evaluated 120 stimulation sequences determining the conduction time between a central dipole (pacing site) and two 15 mm-spaced dipoles. Conduction time intervals were significantly longer in right atrium (RA) than in left atrium (LA). The mean activation time at both atria varied 10±4 ms when the pacing site was 6 mm displaced. When pacing voltage was increased from 2 to 10 times the diastolic threshold, a non-significant reduction in conduction and activation times was observed at both atria. However, no difference was observed in the conduction and activation times when stimulating at two different cycle lengths (300 and 500 ms). According to these findings, the authors postulate that during atrial PM it is not mandatory to stimulate exactly at the same cycle length than the tachycardia to reproduce a similar atrial sequence.

A PM limitation is that pacing at two different points may induce similar surface ECG or endocavitary recordings. An optimal spatial PM resolution requires a short maximum distance between two points generating similar ECG. Usually, the spatial resolution of unipolar stimulation is shorter than 5 mm [3]. Spatial resolution deteriorates with wide electrodes, bipolar stimulation and pacing at pathologic areas.

The highest benefit of PM has been found in focal tachycardia mapping, especially in idiopathic VT. However, PM has been reported in a broad variety of arrhythmias. In the following sections we will review its usefulness and feasibility sorted by type of arrhythmia.

## Atrial Tachycardia

**Figure 1:**
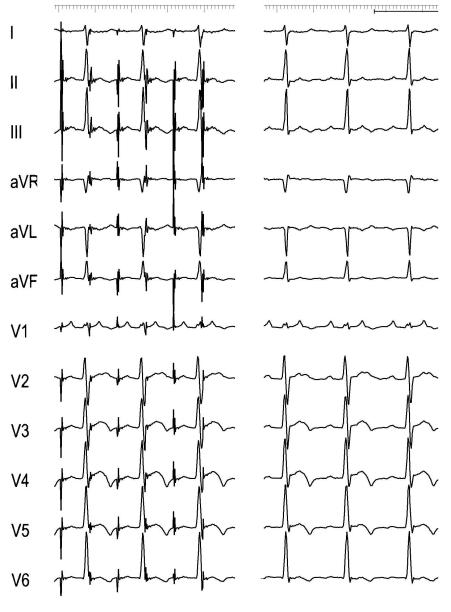
Atrial tachycardia pacemapping. Atrial pacing to emulate the P waveform of the tachycardia

Atrial tachycardia (AT) is defined as focal when its source is a precise point at the atria. Focal AT are due to triggered activity, increased automatism or microreentry. The precise origin of a focal AT can be readily determined using PM by pacing at different sites of the atria to emulate the P waveform of the tachycardia (Figure 1) or the atrial activation sequence during tachycardia. However, proper interpretation of discrete changes in P wave shape is limited by its low voltage and distortion or masking by prior ventricular repolarizations.

McLean et al [5] reported several electrocardiographic criteria to help locating an AT origin. According to these authors, negative P waves in lead I suggest a left atrial origin, specifically an inferior pulmonary vein or coronary sinus (CS) when the P wave is negative or bimodal in lead V1. Generally, pacing close to the CS produces a negative P wave in inferior leads [2]. But Waldo [6] demonstrated that pacing at the upper site of CS generates positive or biphasic P waves in inferior leads. This could be explained by an activation of the LA through the Bachman bundle and later propagation of the excitation wavefront from the top to the bottom across the atria. Yet, Man et al [7] demonstrated that pacing at two different sites, up to 1.7 cm spaced at RA and 3.2 cm spaced at CS, induced similar P wave shapes. These studies confirmed a limited value of surface ECG for PM.

SippensGroenewegen et al [8] used a multiple lead surface ECG (62 leads) to create P wave maps during right atrial pacing at different sites. They found that the 17 evaluated sites showed different activation maps. The RA spatial resolution was 3.5 cm2 in this study; better than the RV spatial resolution (6.7 cm2) previously published [9].

Another tool for targeting AT ablation is PM guided by endocavitary recordings. This technique compares the atrial activation sequence during tachycardia, as shown by endocavitary recordings, and the sequence obtained during pacing with a mapping catheter at different atrial sites. Tracy et al reported excellent results using this technique in 10 focal AT ablation procedures [10].

## Pulmonary Veins

A focal origin has been reported in most patients with paroxysmal atrial fibrillation (AF). These foci are usually located in the pulmonary veins (PV) and its ablation is becoming a curative therapy. Ablation strategies include empirical electric isolation of the four PV or a selective approach ablating only the culprit PV. The latter can be extraordinarily demanding as finding the culprit vein is frequently challenging. Deen et al [11] reported the use of PM to help identify the putative PV. In 10 patients (33 PVs) the authors compared the activation sequences recorded at the crista terminalis (CT) and the CS during selective pacing from the four PVs. CT electrograms were recorded using a 20-pole catheter and a decapolar catheter was inserted in the CS.

Activation patterns at CT and CS differed at every paced PV. In order to measure activation times they considered the CS proximal electrodes (CS 9-10) as a reference, corresponding to time zero. According to their findings they concluded: 1.- Pacing of right PVs showed activation of CS was from proximal to distal: reaching CS 9-10 15.8 ms and 24 ms before CS 1-2 in right superior PV (RSPV) and right inferior PV (RIPV), respectively. Pacing of left PVs showed activation of CS from distal to proximal: reaching CS 1-2 23.8 ms and 20.1 ms before CS 9-10 in left superior PV (LSPV) and left inferior PV (LIPV), respectively. 2.- Activation of CT was earlier in CT 5-6 and CT 11-12 during pacing of  RSPV and RIPV, respectively. Pacing of left PVs showed earlier activation at CT 3-4 with activation pattern from top to bottom. Total activation time of CT was longer for the LSPV (33.7 ms) than for the LIPV (19.3 ms).

An excellent correlation was observed between atrial activation sequence obtained by PM and the location of the focus at the PV under traditional electrophysiological criteria.

## Accesory pathways

Conventional strategies for accesory pathway (AcP) mapping face certain limitations, leading to prolonged procedures and the need for multiple RF applications during ablation. Perez-Castellano et al reported a new mapping approach to help localize the atrial insertion of AcP by reproducing the atrial activation sequence during orthodromic tachycardia through atrial pacing at the mapping catheter [12].

**Figure 2:**
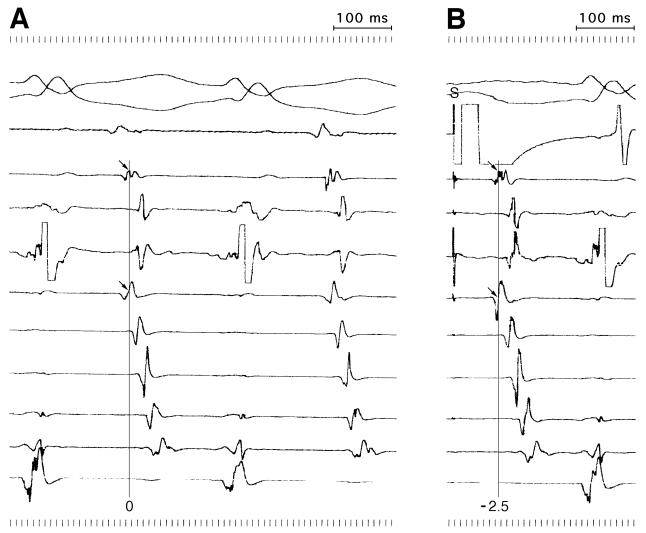
Pacemapping of accesory pathway. Atrial pacing (Panel B)to reproduce the atrial activation sequence during orthodromic tachycardia (Panel A).

This method is based on the relative timing of activation between two stable reference electrograms, used to estimate the atrial activation sequence during tachycardia and during pacing (Figure 2). The authors first estimated the location of the AcP by electrocardiographic criteria. Then, the activation interval between the two atrial references is measured during tachycardia. Atrial pacing is performed at the mapping catheter at different locations closed to the theoretical location of the AcP aiming to reproduce that interval. A difference between the activation time in tachycardia and during pacing lower than 5 ms identified sites with the maximum probability of ablation success. The highest predictive value of success was reached when the following criteria were fulfilled: difference between activation times shorter than 5 msec, V/A local index lower than 1, and a stable local electrogram (EGM).

This method is complementary with traditional mapping criteria [13] and its maximum usefulness is foreseen after non-intentional induction of AcP mechanical block, avoiding procedure discontinuation. A major limitation of the technique is the need for adequate stable references close to the area of interest.

In patients with preexcited tachycardia implicating Mahaim fibers underwent attempted catheter ablation of the accessory pathway is possible to locate the accessory pathway ventricular insertion site used the criteria of concordance between paced and spontaneous QRS morphologies during pace mapping [14]. This criteria is complementary to earliest onset of local electrogram relative to surface preexcited QRS (activation mapping) and a QS-like pattern of unfiltered unipolar electrograms with steep downstroke.

## Idiopathic Ventricular Tachycardia

The right ventricular outflow tract (RVOT) tachycardia is the most frequent idiopatic VT, it origin is usually a single and well-limited focus. In the majority of patients this focus is located in RVOT’s anterior septal wall below the pulmonary valve; occasionally, the posterior septal and free walls are involved. These patients do not show overt structural heart disease, thus ventricular excitation and conduction properties are preserved. These conditions, as opposed to scar tissue, facilitate identifying the origin of the VT using PM.

**Figure 3:**
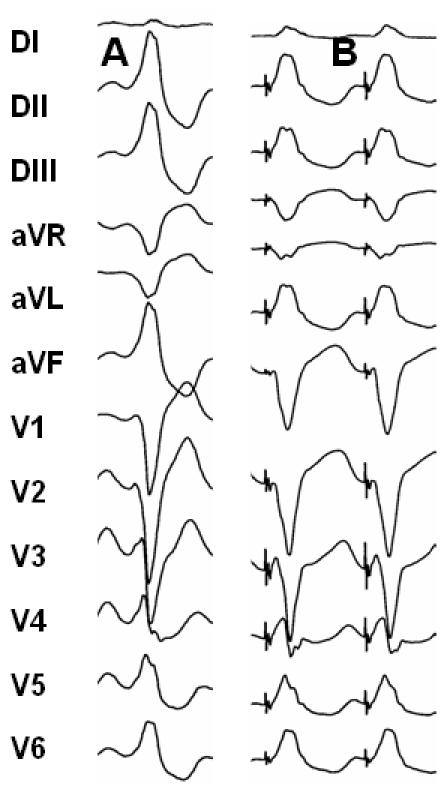
VT pacemapping. Panel A: Left bundle branch block morphology VT, inferior axis and R wave transition in V4.  Panel B: pacing at the right ventricle outflow tract produces a QRS complex similar to the one in tachycardia

Conventionally, ventricular PM is performed in sinus rhythm, pacing at a cycle length similar to the clinical VT, trying to reproduce the QRS morphology during VT (Figure 3). Paced and clinical VT QRS complexes must be similar regarding the pattern of left bundle-branch block, cardiac axis, R/S relationship in precordial leads and any QRS notching. These criteria should ideally be fulfilled in 11 of the 12 leads of the ECG.

Far-field capture may interfere with an adequate PM evaluation by distorting the paced QRS morphology. In order to reduce this detrimental effect, unipolar or bipolar with a distance between poles no longer than 5 mm are the recommended pacing configurations [15]. Regarding the PM spatial resolution in RVOT, Green et al. found similar ECGs pacing at endocardial sites separated up to 8 mm [16].

Activation mapping constitutes an additional tool for idiopathic VT mapping. As an example, in a recently published series of six RVOT tachycardia cases, Timmermans et al using activation mapping located the origin of the tachycardia in the pulmonary artery, followed by successful ablation [17]. Presystolic potentials were registered at the ablation sites. In 3 of these patients pulmonary artery pacing showed an ECG similar to the clinical VT.

Some ECG patterns may suggest the primary location of a VT within the RVOT. The presence of Q waves in DI suggests an anterior septal origin, while R waves in DI suggest a more posterior source. When the R wave transition occurs early in precordial leads suggests an origin in the upper RVOT, a later transition (from V2) points to a lower location, below the pulmonary valve. Digit et al performed PM at the RVOT in healthy individuals in order to define ECG patterns from the different stimulated points to suggest the origin of RVOT tachycardias [18]. The authors reported that tall and narrow R waves on DII suggest a septal origin, while a notch in the R wave on DII and/or a late QRS transition on precordial leads suggest an origin in RVOT free wall. Some limitations of these criteria are a lack of specificity and the possibility of variations according to heart position.

Other idiopathic VT is the fascicular VT, is usually verapamil-sensible and has been demonstrated to arise from the left posterior or left anterior fascicle, with a right bundle branch block configuration and left-axis deviation or right-axis deviation, respectively. The ablation of a fascicular VT can be guided by activation mapping and/or Purkinje potential preceded the QRS during VT, behind VT exit to be associated with an optimal match between the paced rhythm and the clinical VT [19]. Therefore, if fascicular VT is non-inducible the PM is very important for guided the ablation, but ablation at a site with an optimal PM also can be unsuccessful [20].

##  Coronary disease-related VT

Myocardial infarction is the leading substrate for monomorphic sustained VT. In hemodinamically tolerated VT, radiofrequency ablation constitutes a major therapeutic option associated or not to implantable cardioverter defibrillators. Percutaneous VT ablation reduces the rate of recurrences, avoids potential toxic effects of antiarrhythmic drugs and it is associated to a lower risk than EP-guided surgical resection procedures. Gonska et al performing a single morphology VT ablation reported an acute success rate close to 75% [21]. Ablation of multiple morphology VT is associated with lower success rates.

Usefulness of PM in this subset of patients is limited. Scar tissue impairs ventricular conduction properties and favours conduction blockade. Thus, in this context, pacing in sinus rhythm to reproduce an exact VT QRS morphology is usually challenging or unsuccessful.

The electrocardiographic VT morphology is determined by the initial activation site (circuit exit), and by the ventricular activation pathway. When PM is performed at or near the exit of a reentrant circuit, the induced QRS morphology may resemble the VT QRS [22]. However, when pacing is performed within a wide circuit and capture involves distant zones away from its exit, the morphology of QRS may be different despite pacing inside the circuit. Therefore, PM in close zones in these patients may induce significantly different QRS morphologies [22].

As mentioned before, PM spatial resolution worsens with bipolar stimulation by inducing electrical capture at both electrodes. Kadish and coworkers [23] demonstrated that pacing at the same site may induce different QRS morphologies depending on the bipolar or unipolar configuration. Moreover, pacing at 10 mA may induce different morphologies as compared to stimulating at twice the diastolic threshold. Given that the contribution of the proximal electrode (generally anode) to depolarization is variable, Delacretaz et al do not recommend bipolar pacing with separate electrodes for PM or entrainment mapping on post-myocardial infarction VT [24].

PM also helps to characterize slow conduction zones usually located within scars, manifested as a delay between the stimulus and the QRS onset (S-QRS interval). In normal hearts the S-QRS interval is shorter than 40 ms, while most of myocardial areas with abnormal fractionated EGM in sinus rhythm show a S-QRS delay longer than 40 ms. Demonstrating the presence of slow conduction zones is not enough to guide an ablation procedure, as they constitute a regular finding in infarcted areas [24]. However, the presence of abnormal EGMs in sinus rhythm associated with a delay in the S-QRS interval and a similar ECG than the clinical VT, suggests potential key zones to be evaluated by conventional criteria during VT. In cases of fast or poorly tolerated VT, PM may be the primary strategy to guide the ablation procedure.
